# Value of adding 0.01% atropine with orthokeratology for myopia in children: an updated meta-analysis of randomized controlled trials

**DOI:** 10.3389/fped.2025.1571790

**Published:** 2025-06-03

**Authors:** Shudan Tu, Huangfang Ying, Liyang Ni, Zilong Zhang, Weiping Hu

**Affiliations:** Department of Ophthalmology, Affiliated Hospital of Shaoxing University, Shaoxing, Zhejiang, China

**Keywords:** myopia, orthokeratology, atropine, nearsightedness, meta-analysis

## Abstract

**Background:**

This systematic review and meta-analysis aimed to compare outcomes of 0.01% atropine with orthokeratology (AOK) vs. orthokeratology (OK) alone for slowing the progression of myopia in children.

**Methods:**

MEDLINE via PubMed, Embase, Scopus, Web of Science, CENTRAL (Cochrane Central Register of Controlled Trials), Chinese electronic databases of VIP, and Wanfang were searched from inception until 19th August 2024 for randomized controlled trials (RCTs) about the review topic. The primary outcome was a change in axial length (AL) (mm). Secondary outcomes were spherical equivalent refraction (SER) (Diopter), pupil diameter (PD) (mm), amplitude of accommodation (AA) (Diopter), and intraocular pressure (IOP) (mmHg).

**Results:**

10 articles corresponding to eight RCTs were included. Meta-analysis found that change in AL was significantly reduced with AOK as compared to OK alone at 6 months (MD: −0.10 95% CI: −0.14, −0.06 I^2^ = 48%), 12 months (MD: −0.08 95% CI: −0.10, −0.07 I^2^ = 0%) and 24 months (MD: −0.14 95% CI: −0.19, −0.08 I^2^ = 0%). Pooled analysis found that AOK did not reduce the progression of SER (MD: 0.06 95% CI: −0.00, 0.12 I^2^ = 7%) and increased PD (MD: 0.63 95% CI: 0.40, 0.85 I^2^ = 86%) as compared to OK alone. Pooled analysis also found a tendency of reduced AA with AOK as compared to OK alone but without significant results (MD: −0.45 95% CI: −1.00, 0.10 I^2^ = 59%). Meta-analysis failed to show a statistically significant difference in change of IOP between AOK and OK (MD: −0.49 95% CI: −1.48, 0.50 I^2^ = 51%).

**Conclusions:**

AOK seems to be more efficacious in slowing the progression of myopia in children as compared to OK alone.

## Introduction

Myopia is a worldwide public health problem more particularly in eastern Asian countries where the prevalence can reach up to 90% in children. Around 10%–20% of children completing secondary schooling in these areas suffer from sight-threatening pathologies due to myopia (1). Research also indicates that myopia will affect around half of the world's population by 2050 with nearly 10% of the population affected by high myopia (2). Myopia is associated with a significant increase in the risk of pathological changes like glaucoma, cataracts, retinal detachment, and myopic macular degeneration which can lead to permanent loss of vision (3). The economic burden of this disease is also high with estimates indicating US$202 billion per annum (4). Given the widely prevalent problem, appropriate measures slowing the development of myopia in children must be actively researched.

Myopia progresses by axial elongation which can be controlled by optical, pharmaceutical, and behavioral interventions (5). Amongst the available therapies, atropine eye drops and orthokeratology (OK) are the most commonly used, globally (6). OK utilizes a custom-made rigid contact lens which can alter the cornea reducing refractive error and allowing clear unaided vision in daylight (7). Several meta-analysis studies have shown that OK is effective in reducing axial length (AL) in myopic children (8, 9). Likewise, atropine also is found to be effective in slowing the progression of myopia in children. It acts by a direct effect on the globe to reduce eyeball elongation or via an alternate route of relaxing the focusing muscles of the eyes (10). Wei et al. (11) in a meta-analysis of 15 trials have shown that atropine in concentrations of <1% is effective in retarding the diopter and axis growth of myopia in children. Another study has reported that different doses of atropine i.e., low: 0.01%, moderate: 0.01%–0.5%, and high: 0.5%–1% have similar efficacy by adverse effects increase with higher doses. Hence, low-dose atropine (0.01%) should be preferred in clinical practice (12). Given the fact that both OK and atropine are effective in myopia and both have different mechanisms of action, there have been reports of combined treatment with atropine and OK (AOK) to further reduce the progression of myopia in children. Currently, most clinicians still use single interventions for management of myopia but combination therapies are slowly gaining popularity (6). It is also pertinent to mention that atropine is a low cost medication and addition of the same to OK may be not incur high expenditure. If combination therapy is found to be more effective, AOK can be an alternative to OK. Nevertheless, there is also evidence which indicates that AOK can incur substantial indirect and structural costs which can reduce its uptake in some countries (5).

Indeed, the comparative efficacy of AOK vs. OK has been a topic of research for several systematic reviews and meta-analysis studies in the past (13–17). However, the past studies have limitations in including different concentrations of atropine (13, 16, 17), including data from both randomized controlled trials (RCTs) and observational studies (14), and including only a limited number of RCTs (13, 15). Herein, we present the results of the most updated systematic review and meta-analysis examining the efficacy of AOK (with 0.01% atropine) vs. OK alone for managing myopia in children.

## Methods

### Protocol registration and review objective

A protocol of the study approved by all authors was registered on PROSPERO hosted by the National Institute for Health Research, University of York, Center for Reviews and Dissemination. We received the identification number CRD42024579818. We wrote and prepared this manuscript based on the PRISMA reporting guidelines (18).

The purpose of the review was to answer the following clinical question: Does the use of 0.01% atropine in addition to OK improve outcomes of myopia in children?

### PICOS eligibility

The PICOS criteria deemed suitable by the reviewers for including studies was as follows:

1. Patients <18 years of age with myopia. Spherical equivalent refraction (SER) was to be less than −6D at baseline (Population).
2. Comparing AOK (Intervention) with Only OK (Control).
3. Reporting the following Outcomes: Change in AL, SER, pupil diameter (PD), amplitude of accommodation (AA), and intraocular pressure (IOP).
4. Study designs was to be RCTs with minimum follow-up of 6 months.

Exclusion criteria was:

1. Studies using concentrations of atropine other than 0.01%.
2. Non-RCTs.
3. Studies were published only as abstracts and theses.

### Search methods

Literature search was performed on MEDLINE via PubMed, Embase, Scopus, Web of Science, CENTRAL (Cochrane Central Register of Controlled Trials), Chinese electronic databases of VIP, and Wanfang from inception until 19th August 2024. No restrictions were applied regarding language, publication time or location to reveal possible articles. Two reviewers formulated the search strategy and completed the search independently. Search terms were selected for atropine (Atropine, Atropinol, Atropine Sulfate, AtroPen), OK (Orthokeratological Procedure, Orthokeratology, Ortho-K OR, OK lens, Orthokeratology lens) and myopia (Myopia, Myopias, Nearsightedness, Nearsightednesses) to include all possible variations.

Search results from all databases were combined and deduplicated. Two authors then screened the titles/abstracts (if available) of the retrieved studies in the search, in duplicate and independently. Subsequently studies potentially relevant to the review decided based on information in the title and abstract were selected for full-text screening. The full text of an article was retrieved even if one reviewer considered the article potentially relevant. Full-texts of studies were then examined in duplicate and independently by the same reviewers. All discords were resolved via consensus or through settlement by the third reviewer. The reference lists of the included studies were also hand-searched for any other missed RCTs before beginning with data extraction.

### Data extraction and study quality

Two reviewers prepared a table to independently retrieve all relevant information from the included articles. The data extracted included: the name of the first author, publication year, location of the study, study groups, sample size, mean age of participants, baseline axial length and SER, and outcome data. If data pertinent to the quantitative analysis was not reported by a study, the authors contact the corresponding author of the article for information. If no response was received, we omitted the study from the meta-analysis. If multiple records of the same RCT were reported in different studies, we collected all relevant data and analyzed them as a single study or separate studies if the follow-up was different. The primary outcome was the change in AL. Secondary outcomes were: cycloplegic SER, PD, AL, and IOP.

The quality of RCTs was judged by the Cochrane Collaboration risk of bias-2 tool (19). Studies were judged for the randomization process, deviation from intended intervention, missing outcome data, measurement of outcomes, selection of reported results, and overall risk of bias.

### Statistical analysis

Statistical analysis was done on the “Review Manager” (RevMan, version 5.3). Change scores of AL (mm), cycloplegic SER (Diopters), PD (mm), AA (Diopters), and IOP (mmHg) from baseline were pooled for a meta-analysis. Data was extracted as mean and standard deviation (SD). If outcomes were only in graphical form, Engauge Digitizer software was used to extract data. Mean difference (MD) with 95% confidence intervals (CI) were pooled in a random-effects model for all outcomes. A funnel plot was drawn for the primary outcome to examine publication bias. Heterogeneity was checked using chi-square-based Q statistics and the I^2^ statistics. A *p*-value of <0.10 for Q statistic and I^2^ > 50% was indicative of high heterogeneity. Subgroup analysis was conducted based on a follow-up period for the primary outcome. We also conducted a sensitivity analysis for the same by excluding one study at a time and reassessing the results.

## Results

**Figure 1:**
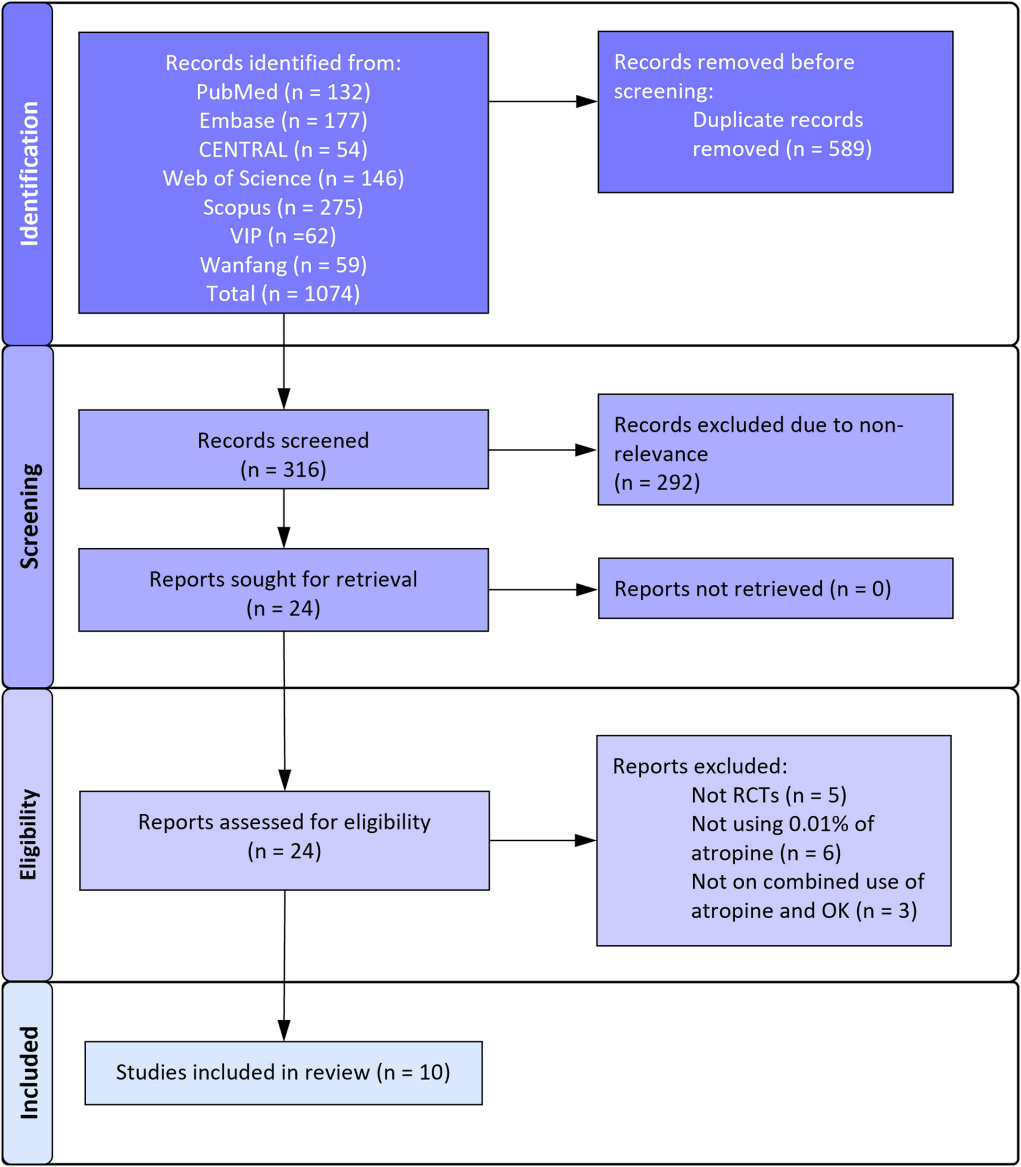
Study flowchart.

Figure 1 depicts the search results as per the PRISMA flowchart. The reviewers found 24 articles to be worth considering for selection. There was no disagreement between reviewers regarding the selection of studies for full-text analysis. Finally, 10 articles (20–29) were found to be eligible for this review and the remaining were excluded for reasons mentioned in Figure 1.

Study characteristics are shown in Table 1. There were 10 articles corresponding to eight RCTs. Yu et al. (21) conducted a double-blinded cross-over RCT and reported the results of baseline and cross-over groups in separate articles (20, 21). Except for one RCT [with two follow-up reports (27, 28)], all others were conducted in China. The combined sample size of all RCTs was 631. The largest RCT was of Shi et al. (29) including 47 patients each. Minimum number of patients in each group was at least 20. The mean age of participants was not reported in three RCTs but included only pediatric cases. In all other studies, the mean age of patients varied around 9–10 years. The follow-up of two studies was only six months. Three trials reported a follow-up of 2 years while all others reported a follow-up of 1 year. Yu et al. (21) mentioned the use of preservatives in the atropine solution while others did not report the information or did not use preservatives.

**Table 1:** Details of included studies.

Author	Location	Groups	Sample size	Mean age (years)	SER, D	Axial length, mm	Maximum follow-up (months)	Axial length measurement
Shi 2017	Shiyinghui Eye Clinic, China	AOK OK	47 47	NR	NR	24.75 ± 0.14 24.87 ± 0.22	6	IOL master
Kinoshita 2018	Konno Eye Clinic or Omiya Hamada Eye Clinic, Japan	AOK OK	20 20	10.9 ± 1.4 10.4 ± 1.9	−2.81 ± 1.43 −2.95 ± 1.43	24.73 ± 0.58 24.95 ± 0.92	12	IOL master
Kinoshita 2020	Konno Eye Clinic or Omiya Hamada Eye, Japan	AOK OK	38 35	10.3 ± 1.6 10.4 ± 1.7	−2.6 ± 1.29 −2.72 ± 1.31	24.69 ± 0.58 24.86 ± 0.81	24	IOL master
Vincent 2020	The University of Hong Kong, China	AOK OK	25 28	8.9 ± 1.2 9.1 ± 1.1	−2.38 ± 0.81 −2.58 ± 0.91	24.38 ± 0.62 24.44 ± 0.84	6	IOL master
Tang 2020 (group 1)	First Affiliated Hospital of Chengdu Medical College, China	AOK OK	20 22	NR	−2.56 ± 1.15 −2.59 ± 1.12	23.72 ± 0.31 23.7 ± 0.29	12	IOL master
(group 2)		AOK OK	43 41	NR	−4.9 ± 1.16 −4.92 ± 1.21	24.69 ± 0.34 24.71 ± 0.37	12	IOL master
Zhao 2022	Second Affiliated Hospital of Dalian Medical University, China	AOK OK	20 21	10.9 ± 1.3 11 ± 1.2	−2.85 ± 0.45 −2.75 ± 0.46	24.56 ± 0.39 24.42 ± 0.48	13	LS−900
Yu 2022	First Affiliated Hospital of Zhengzhou University, China	AOK OK	27 26	10.1 ± 1.4 9.8 ± 1.6	−2.81 ± 0.92 −2.81 ± 0.97	24.79 ± 0.72 24.64 ± 0.79	12	IOL master
Tan 2023	School of Optometry of The Hong Kong Polytechnic University, China	AOK OK	34 35	9.2 ± 1 9.1 ± 1.2	−2.76 ± 0.88 −2.83 ± 1.01	24.56 ± 0.71 24.5 ± 0.92	24	IOL master
Xu 2023	Sun Yat-­ Sen University, China	AOK OK	42 40	10.3 ± 1.1 10.1 ± 1.5	−3.1 ± 1.6 −3.05 ± 1.13	−3.1 ± 1.16 −3.05 ± 1.13	24	LS−900
Li 2024 (cross-over results of Yu 2022)	First Affiliated Hospital of Zhengzhou University, China	AOK OK	26 26	9.8 ± 1.6 9.9 ± 1.5	−2.81 ± 0.97 −2.77 ± 0.89	24.64 ± 0.79 24.71 ± 0.79	12	IOL master

SER, spherical equivalent refraction; AOK, atropine plus orthokeratology; OK, orthokeratology only; NR, not reported; NA, not available.

Details of the risk of bias analysis are shown in Table 2. Five studies had a low risk of bias across domains. One trial was found to have a high risk of bias. Three remaining trials were found to have some concerns. We marked studies with “some concerns” as there was no clarity on the exact details of randomization and blinding of outcome assessment in the studies marked so.

**Table 2:** Risk of bias analysis.

Author	Randomization process	Deviation from intended intervention	Missing outcome data	Measurement of outcomes	Selection of reported result	Overall risk of bias
Shi 2017	Some concerns	Low risk	Low risk	Some concerns	Low risk	High risk
Kinoshita 2018	Low risk	Low risk	Low risk	Low risk	Low risk	Low risk
Kinoshita 2020	Low risk	Low risk	Low risk	Low risk	Low risk	Low risk
Vincent 2020	Some concerns	Low risk	Low risk	Low risk	Low risk	Some concerns
Tang 2020	Low risk	Low risk	Low risk	Some concerns	Low risk	Some concerns
Zhao 2022	Some concerns	Low risk	Low risk	Low risk	Low risk	Some concerns
Yu 2022 & Li 2024	Low risk	Low risk	Low risk	Low risk	Low risk	Low risk
Tan 2023	Low risk	Low risk	Low risk	Low risk	Low risk	Low risk
Xu 2023	Low risk	Low risk	Low risk	Low risk	Low risk	Low risk

### Change in Al

**Figure 2:**
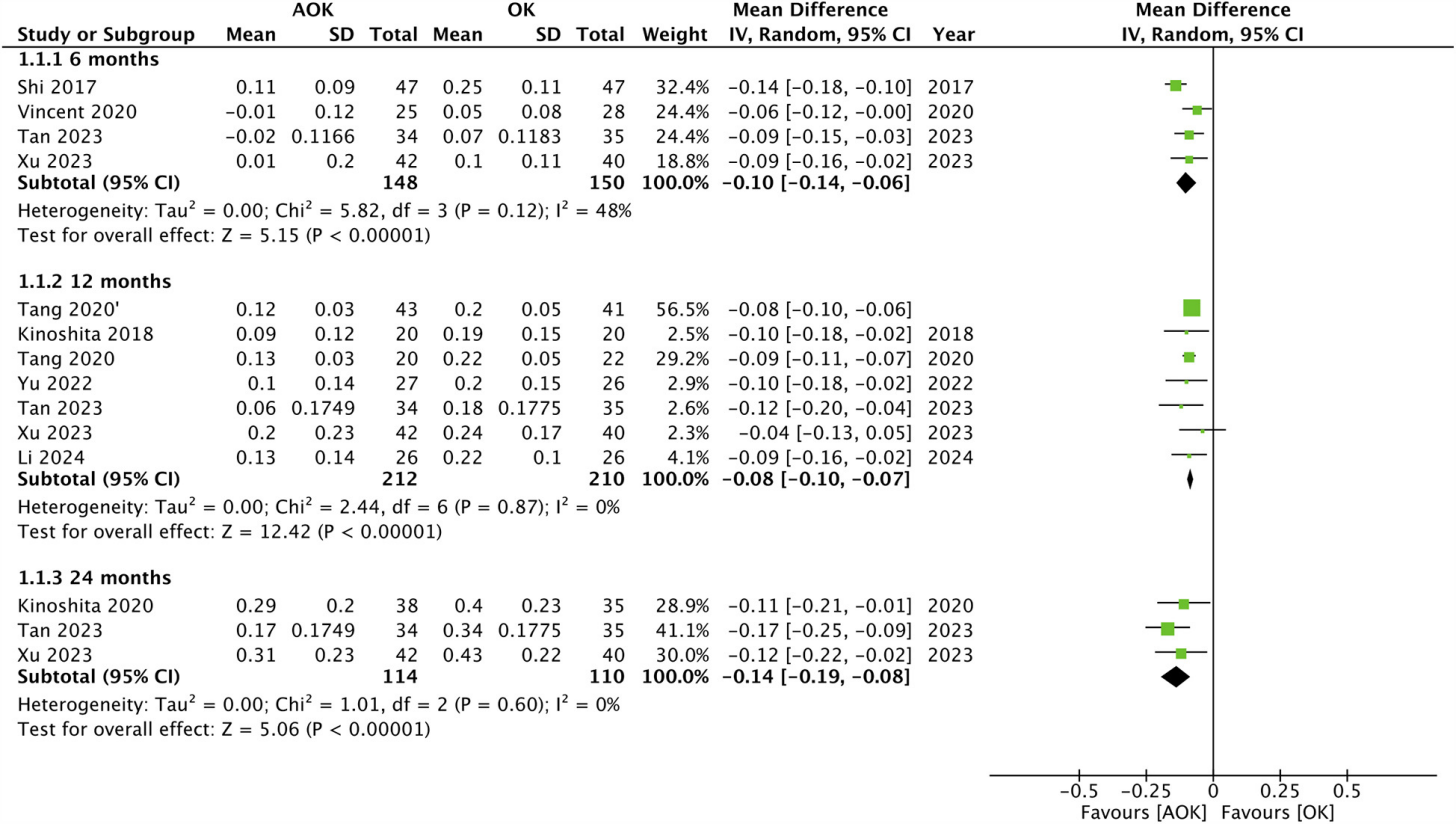
Meta-analysis of change in AL between AOK and OK groups with subgroup analysis based on follow-up period.

Changes in AL scores were reported by the maximum number of included studies. Data was segregated based on the length of follow-up. The pooled analysis is presented in Figure 2. Five studies reported data after 6 months. Meta-analysis found that change in AL was statistically significantly lower with AOK as compared to OK alone (MD: −0.10 95% CI: −0.14, −0.06 I^2^ = 48%). Eight studies constituting nine groups reported 12-month data. Here again, the pooled analysis showed that change in AL was statistically significantly reduced with AOK as compared to OK alone (MD: −0.08 95% CI: −0.10, −0.07 I^2^ = 0%). Only three studies reported data after 24 months of follow-up. Meta-analysis again showed that change in AL was statistically significantly reduced with AOK as compared to OK alone (MD: −0.14 95% CI: −0.19, −0.08 I^2^ = 0%). Sensitivity analysis showed that outcomes were robust for all follow-up intervals.

### Secondary outcomes

**Figure 3:**
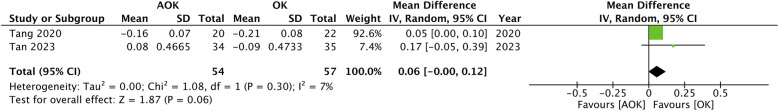
Meta-analysis of change in SER between AOK and OK groups.

**Figure 4:**
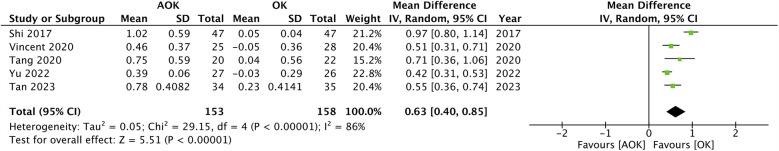
Meta-analysis of change in PD between AOK and OK groups.

**Figure 5:**
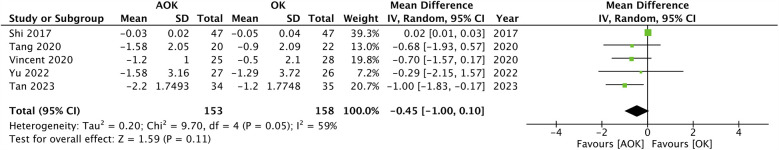
Meta-analysis of change in AA between AOK and OK groups.

**Figure 6:**
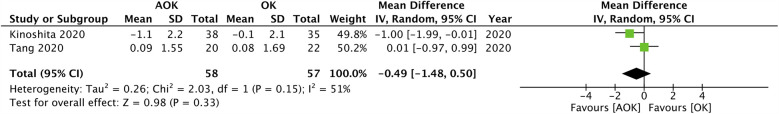
Meta-analysis of change in IOP between AOK and OK groups.

Only two studies with 111 participants reported a change in SER scores. The pooled analysis found that the progression of SER was not significantly different between AOK as compared to OK alone (MD: 0.06 95% CI: −0.00, 0.12 I^2^ = 7%) (Figure 3). Five studies with 311 participants reported data on PD. Meta-analysis showed that AOK significantly increased PD as compared to OK alone (MD: 0.63 95% CI: 0.40, 0.85 I^2^ = 86%) (Figure 4). Data on AA was reported by five trials with 311 patients. Pooled analysis found a tendency of reduced AA with AOK as compared to OK alone but without significant results (MD: −0.45 95% CI: −1.00, 0.10 I^2^ = 59%) (Figure 5). Change in IOP was mentioned in two studies only (115 patients). Pooled analysis failed to show statistically significant results between AOK and OK (MD: −0.49 95% CI: −1.48, 0.50 I^2^ = 51%) (Figure 6).

## Discussion

The current study is the most updated meta-analysis of only RCTs comparing outcomes of 0.01% AOK vs. OK alone in slowing the progression of myopia in children. A detailed literature search revealed 10 articles which included reports of eight RCTs, all from Asian countries. The baseline age and SER were more or less similar across the included RCTs. Meta-analysis of all eight RCTs revealed that combination treatment with AOK resulted in a significant reduction in AL at 6 months, 12 months, and 24 months of follow-up as compared to OK alone. Importantly, the MD at 24 months was only slightly higher than the MD of change in AL noted at 6 months. One reason for this could be that only a limited number of studies have reported 2-year results. Secondly, it is also postulated that low-dose atropine can lead to temporary choroidal thickening in young children. Also, a combination treatment of AOK can lead to more thickening than OK alone (30, 31). The fundamental mechanism of choroidal thickening with atropine administration remain ambiguous. Research indicates that nitric oxide may contribute to the choroidal thickening caused by atropine, potentially via affecting blood flow and the stromal elements of the choroid through the relaxation of both vascular and nonvascular smooth muscles in the choroid. Furthermore, dopamine may potentially contribute to the choroidal thickening elicited by atropine. Intravitreal injection can enhance dopamine release from the retina, and D2 agonists have been shown to augment choroidal thickness in animal models utilizing negative lenses (30, 31). A study also indicates that choroidal thickness has a reverse association with axial length in myopic children (32). Therefore, the decreased AL in the first year with AOK could be due to a temporary, slight choroidal thickening after using atropine, thus exaggerating the axial elongation control effect (33). Research also shows that the efficacy of OK is most in the first 12 months of treatment and reduced with longer use (34). This could be another possible reason for the diminished efficacy of AOK by 24 months. Despite this minor anomaly, the results of change in AL were stable on sensitivity analysis demonstrating the robustness of the results. The lack of publication bias also adds to the credibility of the results, thereby demonstrating that the outcomes are reliable and can be applied in clinical practice. Secondary outcomes were reported by a limited number of studies. Due to this reason, a subgroup analysis based on treatment time was not possible and data from the maximum follow-up was used. Only two studies reported change in SER which showed a borderline non-significant result. A similar significant result with SER can be expected when there is a significant difference in AL between the two groups. However, the number of studies reporting data on AL and SER were vastly different. Majority studies reported data on AL but only two studies were available for the meta-analysis on SER which could have contributed to the non-significant results. Regarding other important safety outcomes, there was no change in IOP and AA. The meta-analysis showed that PD increased with AOK as compared to OK alone.

There have been previous meta-analyses published in recent years which also demonstrate similar outcomes as our meta-analysis but with important limitations. Gao et al. (13) in a meta-analysis of five studies (three RCTs and two observational studies) have also reported a significant reduction in AL with 0.01% AOK as compared to AOK alone in myopic children. Other than the reduced number of RCTs, their review could not assess other outcomes in the meta-analysis. Wang et al. (15) in 2021 published their meta-analysis of four RCTs which too concurred with the current results. However, only two studies in their review reported follow-up data of 12 months. A meta-analysis by Yang et al. (17) combining RCTs and observational studies (total of eight) also found that combining low-dose AOK results in better outcomes as compared to AOK alone. The meta-analysis of Zheng et al. (33) also combined all study types (10 RCTs and five observational studies) and articles with different concentrations of atropine to demonstrate the superiority of AOK vs. OK in myopic children. By far the largest meta-analysis has been that of Wang et al. (14) which specifically examined the same research question as our review. However, they too combined RCTs and observational studies and included the same RCTs (27, 28, 35, 36) with different follow-up times repeatedly in the same meta-analysis thereby generating erroneous results. We not only included four new articles (20–23) but also excluded retrospective studies and corrected the errors of their review to present the best possible evidence on the efficacy of 0.01% AOK vs. OK alone for treating myopia in children.

While the current review provides pooled evidence on the effectiveness of AOK, there was important heterogeneity in the meta-analyses, especially for the secondary outcomes. This could be due to the methodological variations amongst studies regarding the baseline study population, baseline SER, material and design of the OK lens, and follow-up intervals. Importantly, the most important outcome of change in AL at 12 and 24 months had no inter-study heterogeneity which provides reassurance on the applicability of the results. The exact mechanism behind the increased efficacy of AOK vs. OK is unclear. OK is postulated to induce myopia by defocusing on the peripheral retina by altering the corneal shape, moderating eye growth and hypermyopia (37, 38). Human studies show that OK can cause defocusing of myopia by reducing the central curvature and increasing the peripheral curvature of the cornea (39, 40). Given the fact that both groups used OK, the better efficacy can be attributed to the effects of atropine. The addition of atropine may have resulted in improvement of peripheral defocus in myopia control. Research shows that the effectiveness of the OK lens in reducing AL could be enhanced by higher PD (41). Furthermore, larger PD may have caused a myopic shift in the peripheral retina thereby improving retinal illumination.

The degree of baseline myopia is a very important confounding factor that could not assessed in the current review due to the significant overlapping of the SER range amongst the included studies. Kinoshita et al. (27, 28) in their trial noted that the synergistic effect of AOK in reducing AL was more in low myopia cases while both AOK and OK had similar efficacy in high myopia. On the other hand, Xu et al. (22) in their trial noted that baseline SER did not affect the efficacy of AOK or OK therapy. It is also pertinent to note that the defocus on the peripheral retina is further enhanced as the magnitude of myopia correction by OK therapy increases, moving from hyperopic to myopic defocus. Consequently, the defocus on the peripheral retina in participants undergoing OK monotherapy with high SER at enrollment may have been adequately ameliorated, whereas those with a low SER may not show similar improvement. It can be postulated that the addition of atropine to OK is more efficacious via this mechanism in individuals with a low baseline SER, as the defocus on the peripheral retina was inadequately ameliorated by OK monotherapy (27, 28). Given the limited data in the literature, a more thorough analysis of the efficacy of AOK is needed in high vs. low myopia groups.

There are limitations to this review. Firstly, the quality of all included RCTs was not high. Several trials had bias or concerns regarding the randomization process and blinding of outcome assessment. Zhao et al. (24), Vincent et al. (26) and Shi et al. (29) had some concerns regarding the randomization process while Tang et al. (25) and Shi et al. (29) had concerns regarding blinding of outcome assessment. Secondly, all trials were from Asian countries and no data was available from Western populations. Given the fact that ethnic variations exist in the efficacy of AL reduction by interventions (42), the results should not be generalized till studies from Western populations are reported. Thirdly, due to the limited reporting of data by the included studies, we were unable to assess other important outcomes like tear film break-up time, choroidal thickness, and corneal endothelial cell density. Fourthly, the number of patients included in the trials was not high and this may have affected the statistical power of our analysis. Fifthly, there was high inter-study heterogeneity for the secondary outcomes. Given the small number of studies, we could not examine the source of such heterogeneity by subgroup analysis, hence, these results must be interpreted with caution. Lastly, only a few trials reported long-term 2-year data. Further studies are needed to establish the long-term efficacy of AOK over OK alone.

## Conclusions

The results of this updated meta-analysis of only RCTs indicate that combination therapy of 0.01% AOK results in significantly better AL control as compared to OK alone in myopic children. PD may increase with combination therapy but it may not affect AA and IOP.
